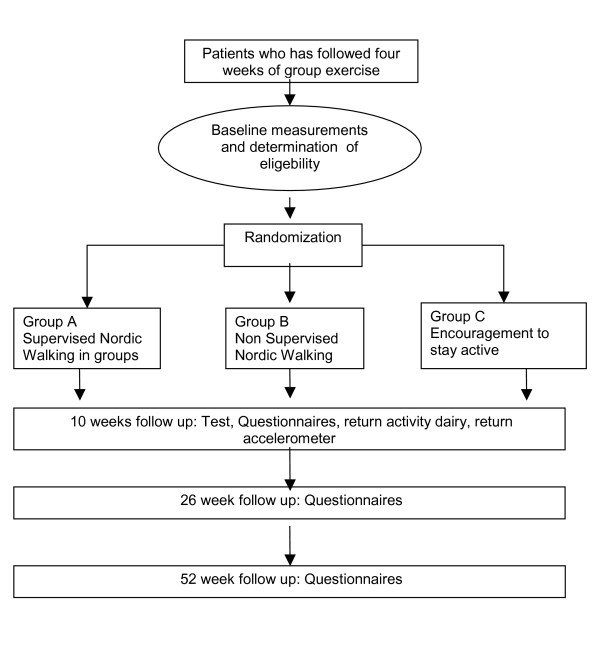# Nordic Walking and chronic low back pain: design of a randomized clinical trial

**DOI:** 10.1186/1471-2474-7-77

**Published:** 2006-10-02

**Authors:** Lars Morsø, Jan Hartvigsen, Lis Puggaard, Claus Manniche

**Affiliations:** 1 Clinical Locomotion Science, Institute of Sports Science and Clinical Biomecanics, University of Southern Denmark, Odense, Denmark; 2 Back Research Center, Hospitals of Funen, University of Southern Denmark, Ringe, Denmark; 3 Nordic Institute of Chiropractic and Clinical Biomechanics, Odense, Denmark; 4 ACES, Institute of Sports Science and Clinical Biomechanics, University of Southern Denmark, Odense, Denmark

## Abstract

**Background:**

Low Back Pain is a major public health problem all over the western world. Active approaches including exercise in the treatment of low back pain results in better outcomes for patients, but it is not known exactly which types of back exercises are most beneficial or whether general physical activity provide similar benefits.

Nordic Walking is a popular and fast growing type of exercise in Northern Europe. Initial studies have demonstrated that persons performing Nordic Walking are able to exercise longer and harder compared to normal walking thereby increasing their cardiovascular metabolism. Until now no studies have been performed to investigate whether Nordic Walking has beneficial effects in relation to low back pain.

The primary aim of this study is to investigate whether supervised Nordic Walking can reduce pain and improve function in a population of chronic low back pain patients when compared to unsupervised Nordic Walking and advice to stay active. In addition we investigate whether there is an increase in the cardiovascular metabolism in persons performing supervised Nordic Walking compared to persons who are advised to stay active. Finally, we investigate whether there is a difference in compliance between persons receiving supervised Nordic Walking and persons doing unsupervised Nordic Walking.

**Methods:**

One hundred and fifty patients with low back pain for at least eight weeks and referred to a specialized secondary sector outpatient back pain clinic are included in the study. After completion of the standard back centre treatment patients are randomized into one of three groups: A) Nordic Walking twice a week for eight weeks under supervision of a specially trained instructor; B) Unsupervised Nordic Walking for eight weeks after one training session with an instructor; C) A one hour motivational talk including advice to stay active. Outcome measures are pain, function, overall health, cardiovascular ability and activity level.

**Results:**

No results available at this point.

**Discussion:**

This study will investigate the effect of Nordic Walking on pain and function in a population of people with chronic LBP.

**Trial Registration:**

registration # NCT00209820

## Background

Low back pain (LBP) is a major public health problem all over the western world and one of the leading causes for sick leave and disability [1]. In Denmark, 35–50% of the adult population have suffered from LBP, in the last year, and 21% have had symptoms the past 14 days [2]. Therefore high quality research aiming at identifying effective treatment and prevention strategies is warranted.

Throughout the last three decades the management of LBP has changed radically from primarily passive treatments and bed rest to patient centred active treatments involving exercise, resulting in improved outcomes with regards to pain, daily function, and return to work [3-5]. As a result of this development, a wide variety of specific exercise regimes have emerged, however, there is little evidence that one type of exercise is superior to another in the treatment of LBP, even though sub-groups of patients benefiting from specific exercise interventions have been identified [5]. Based on results from a recent randomized clinical trial Hurwitz et al. went as far as to suggesting that maybe LBP patients should refrain from back specific exercises and instead focus on non-specific physical activities in order to reduce pain and improve health [6], thereby adding LBP to the already long list of health conditions that are influenced positively by physical activity.

Nordic Walking (NW) is a fast growing and popular way to exercise in northern Europe, in particular in Scandinavia and Germany [7]. NW is low-tech and cheap; it doesn't require a lot of skill and can be done by almost anybody. NW is performed by walking using poles with rubber or spike tips in the same way as in Nordic style skiing. By using the poles the muscles in the upper body are activated and the length of each step taken is increased resulting in a faster gait. Consequently, NW appears to increase cardiovascular metabolism even in healthy people [8,9]. So far the effect of NW on pain and disability in relation to LBP has not been investigated however if shown effective, NW would constitute an attractive low-tech, low-risk, and low-cost intervention. Furthermore, it is not known whether LBP patients are willing to comply with the prescribed dose and frequency, a problem commonly encountered in other forms of exercise therapy for LBP [10], and whether compliance plays a role in relation to pain and disability outcomes.

The primary aim of this single blind randomized clinical trial is therefore to compare the effect of supervised NW versus non-supervised NW versus information about the benefits of physical activity in relation to pain and pain related function using standardized outcome measures. Secondary objectives are 1) to compare the increase in cardiovascular metabolism between the supervised NW group and the information group and 2) to evaluate compliance to NW between the supervised and non-supervised NW group.

## Methods

### Design

Singleblinded Radomized Clinical Trial.

### Study sample

The patients are recruited from a secondary sector specialized out-patient back pain clinic. All participants receive information about self-care for back pain and attend group exercises twice a week for four weeks before being offered inclusion into the current project. 50 patients are to be included in each of the three intervention groups, totalling 150 participants.

### Inclusion/exclusion

To be included in the project participants must

• have had LBP with or without leg pain > 8 weeks

• have received four weeks of treatment in the primary sector by a family physician, chiropractor, physical therapist, or a combination thereof

• have ended all examinations, individual and group treatment at the back clinic with at least a 75% attendance rate

• be able to read and understand Danish

Exclusion criteria are

• pain less than 3 on the 11 point numeric rating scale

• co-morbidity preventing patient from participating in the full intervention

• unable to sit on a stationary bike for at least 30 minutes in order to perform watt max bicycle test

### Examination and baseline data

One week after ending the group exercise program, potential participants are assessed in an individual baseline test session. First, they complete a battery of questionnaires including information on social, psychological, occupational, educational, physical and lifestyle factors, expectations to treatment outcome, and baseline values for the outcome measures (see below). Finally, each participant performs a watt max cycle test in order to evaluate their cardiovascular capacity.

### Randomization

Randomization is carried out by a project secretary blinded to the interventions. Participants consenting to participate draw a sealed envelope containing information about treatment allocation. Envelopes are arranged in clusters of 15 to secure an even spread in all groups.

### Interventions

After test session and randomization, participants are allocated to one of three groups

**Group A **is instructed and performs NW in groups of 6–8 twice a week for 8 weeks under supervision of a specially trained instructor. To monitor the activity level in this group accelerometers are provided for the participants. The participants have to use accelerometers through out the day for 2 weeks (week 4 and 5) during the intervention. In the same period they have to fill out an exercise dairy describing their daily activity.

**Group B **is instructed in NW once by a specially trained instructor, but is afterwards told to perform NW as much as they want to at home on their own for the next 8 weeks. This group also reviews accelerometers and an exercise dairy for 2 weeks (week 4 and 5 during the intervention.

**Group C **is given standard information about exercise and maintaining the daily function level they have achieved during the treatment at the back pain clinic.

NW poles are provided free of charge to everyone included in the project. Participants randomized to group C received their poles after completion of the 8 weeks.

### Outcome measures

#### Primary outcome measures

##### Low Back Pain Rating Scale

Low Back Pain Rating Scale was developed to measure the dimensions of pain, disability, and physical impairment for patients with LBP [11]. The pain assessment index is measures using three 11-box numeric rating scales (pain now, worst and average pain in the last two weeks) for back and leg pain separately. Each response scale score is added giving a scale range of 0–60 points. The disability index comprises 15 items scaled as yes = 0 points, can be a problem = 1 point, no = 2 points, giving a total of 0–30 points

##### Patient Specific Function Scale

Patient Specific Function Scale was developed to assess functional limitations in a variety of clinical presentations. Patients are asked to identify three important activities with which they are having difficulty or are unable to perform because of their problem. In addition to specifying the activities, patients are asked to rate on an 11-box numeric rating scale the current level of difficulty associated with each activity [12]

#### Secondary outcome measures

##### EQ-5D

EQ-5D is a standardized 5-item generic measure of health related quality of life. Domains of mobility, self-care, usual activities, pain/discomfort, and anxiety are assessed using a three point response scale [13].

##### Medication use, other treatment for LBP, time off work

Information collected using questionnaires at different time points.

Outcome measures used in relation to objective number two (increase in cardiovascular metabolism).

##### Watt Max Bicycle Test

Watt Max Bicycle Test is a standardized test to determinate a persons cardiovascular capacity [14]. The test person rides a stationary exercise bike and resistance I gradually increased every three minutes until the test person reaches the point of excursion.

Outcome measures used to collect information about compliance

##### Accelerometers

Studies have shown that physical activity can be monitored by accelerometers with good reliability and validity [15-17]. In the current project the participants wear the accelerometers the fourth and fifth week of the intervention. The accelerometer (actigraph GT256) is sent by mail, with a detailed instructions about correct use. At the end of the two week data collection period, participants return the accelerometer by mail and data are electronically downloaded and stored.

##### Exercise dairy

The activity level is also measured subjectively using a self-completed activity booklet. The booklet, which is filled out in the same two weeks as the accelerometer is worn, has previous been used in studies where subjective information from participants regarding their level of activity has been collected [18]. Data from the booklet will be compared with data from the accelerometer.

### Follow-up

Follow-up data are collected at eight weeks, six months and one year (Figure 1).

### Data analyses

Power is calculated based on the primary outcome measures. A sample size of 150 participants will provide 80% power to detect a difference of one unit on the NRS (SD = 2.0) between the experimental and control group, assuming alpha of 0.05. This allows for loss to follow up of 20%.

Data will be analyzed by a research group member (JH) blinded to group status. The analysis will be based on intention to treat principles. Both parametric and non parametric principles will be used to compare treatment effects between the groups to identify baseline predictors for successful treatment outcome. Finally, based on a prior definition of success, numbers needed to treat will be calculated.

### Approval

The study is approved by the regional ethics committee for Funen and Vejle Counties, approval # VF 2005005

## Discussion

This is a presentation of a RCT dealing with the effect of Nordic Walking on pain and functional limitations in a population of chronic LBP patients. The inclusion period is expected to last throughout 2006, and results of the trial will be presented as soon as they are available.

## Competing interests

The author(s) declare that they have no competing interests.

## Authors' contributions

LM is the study manager and was involved in the design of the study, wrote the first draft for the manuscript and participated in the subsequent revisions. JH secured funding for the study, participated in the design of the study and was the primary person in revision of the manuscript. LP participated in the design of the study, in particular with respect to the measurements of physical activity and cardiovascular outcome, and commented on the manuscript drafts. CM participated in the design of the study and commented on the manuscript.

## Pre-publication history

The pre-publication history for this paper can be accessed here:



## Figures and Tables

**Figure 1:**